# A method for characterizing the thermal stability and antimicrobial binding to Lipopolysaccharides of Gram-negative isogenic mutant strains

**DOI:** 10.1016/j.mex.2021.101474

**Published:** 2021-07-30

**Authors:** Belén Navarro, Mackarenna Alarcón, Maricarmen Osees, Felipe Gómez-Alvear, Romina V. Sepúlveda, Jaime Huerta, María Cecilia Opazo, Daniel Aguayo

**Affiliations:** a Center for Bioinformatics and Integrative Biology (CBIB), Facultad de Ciencias de la Vida, Universidad Andres Bello, Santiago 8370146, Chile; b Interdisciplinary Center for Neuroscience of Valparaíso, Faculty of Science, University of Valparaíso, Valparaíso 2340000, Chile; c Instituto de Ciencias Naturales, Facultad de Medicina Veterinaria y Agronomía, Universidad de las Américas, Santiago, Chile; d Millennium Institute of Immunology and Immunotherapy

**Keywords:** Differential Scanning Calorimetry (ITC), Isothermal Titration Calorimetry (ITC), Antimicrobial binding, Lipopolysaccharides

## Abstract

Isothermal Titration Calorimetry (ITC) is widely employed to assess antimicrobial affinity for lipopolysaccharide (LPS); nevertheless, experiments are usually limited to commercially available-LPS chemotypes. Herein we show a method that uses Differential Scanning Calorimetry (DSC) to characterize homogeneity artificial vesicles of LPS (LPS-V) extracted from isogenic mutant bacterial strains before analyzing the antimicrobial binding by ITC. This method allows us to characterize the differences in the Polymyxin-B binding and gel to crystalline liquid (β↔α) phase profiles of LPS-V made of LPS extracted from *Escherichia coli* isogenic mutant strains for the LPS biosynthesis pathway, allowing us to obtain the comparable data required for new antimicrobial discovery.

A method for:

• Obtaining LPS vesicles from isogenic mutant bacterial strains.
• Characterize artificial LPS vesicles homogeneity.
• Characterize antimicrobial binding to LPS.

## Graphical Abstract

**Figure:**
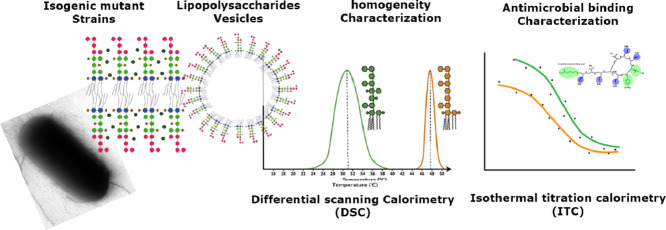

Specifications Table

**Table:** 

Subject Area:	Biochemistry, Genetics and Molecular Biology
More specific subject area:	*Microbiological biophysics, antimicrobial binding*
Method name:	Calorimetric methods to study Lipopolysaccharide-vesicles
Name and reference of original method:	*NA*
Resource availability:	*See Tables ST3 and ST4.*

**Method details**

Lipopolysaccharides (LPS) are the main constituent of the outer membrane (OM) of Gram-negative bacteria and act as a physical barrier against exogenous compounds. Each LPS molecule is composed of a hydrophobic region called lipid A endotoxin, a joining core oligosaccharide (OS), and the hydrophilic region formed by the glycan O-antigen (Fig. 1A) [1]. The LPS amphiphilic character allows them to self-assemble into micelles and vesicles (LPS-V), which are used to understand how antimicrobials pass through the outer membrane or interact with chemical moieties of the LPS. Several LPS-chemotypes can be obtained from commercial companies, which restraint conclusions when biophysical assays are compared with microbiological assays. Herein we show a protocol that uses LPS extracted from isogenic mutant bacterial strains usually employed in the microbiological laboratory to solve this. As an example, we use these LPS to generate composition- and size-controlled vesicles to characterize their structural effect on the LPS-layer fluidity and into an antimicrobial binding by calorimetric methods, such as Isothermal Titration Calorimetry (ITC) and Differential Scanning Calorimetry (DSC) [2].

**Fig. 1:**
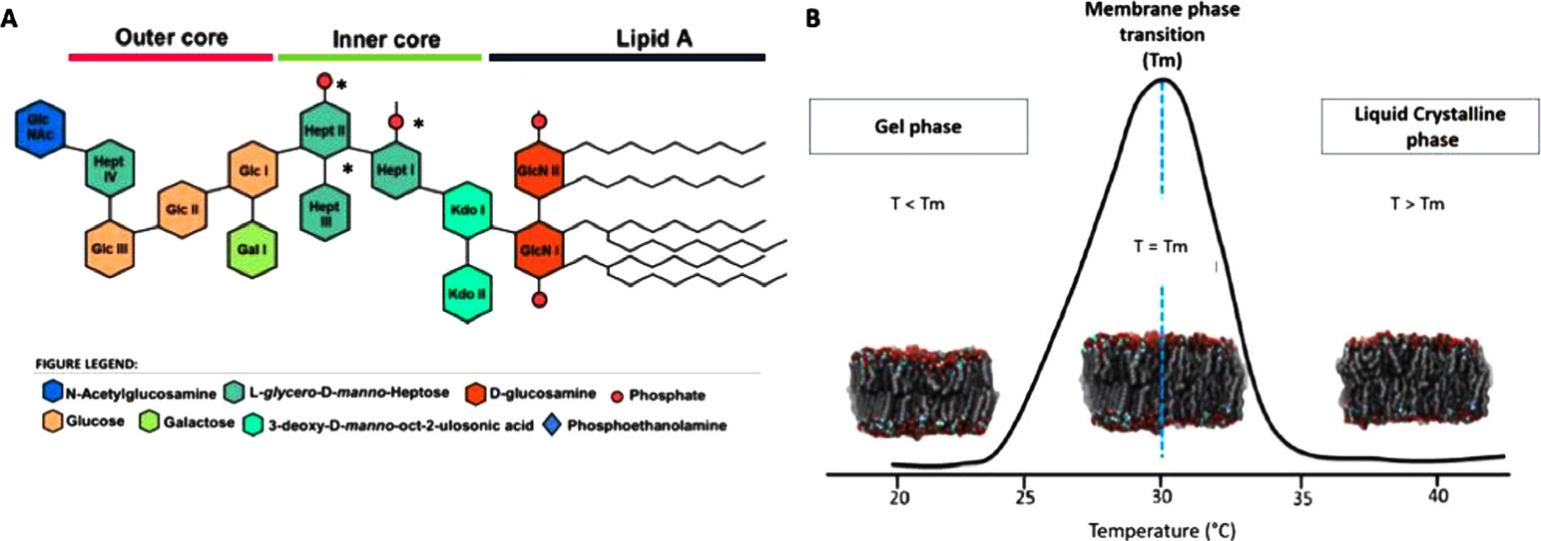
A) Schematic structure of *E. coli* BW25113 *wild-type* and Δ*waaP* LPS. Groups with * are absent in the Δ*waaP* LPS chemotype. B) Lipid membrane phase transition diagram as observed by DSC. Gel to crystalline liquid (β↔α) phase profiles [3].

Isothermal titration calorimetry and DSC provide quantitative and qualitative data on endothermic and exothermic processes, which characterize drug binding and penetration modes to Gram-negative bacteria, providing highly valuable information for new antibiotic discovery. Modern ITC and DSC equipment have higher sensitivity and lower volume cells; nevertheless, relatively high analyte concentrations are required to measure the heat transferred across calorimetric experiments properly. This research method describes a protocol for low volume ITC and DSC equipment to characterize artificial LPS-V antimicrobial binding isotherms and vesicles thermograms. This method allows characterizing the differences in the Polymyxin-B binding process and gel to crystalline liquid (β↔α) phase profiles of the LPS-V made with LPS-chemo-types extracted from mutant bacterial strains (Fig. 1B).

## Experimental procedures

### Lipopolysaccharide extraction & quantification

The method presented herein corresponds to a modification of the hot phenol-water LPS extraction method described by Marolda and Davis [4,5]. In brief, centrifuge 200 mL of *E. coli* BW25113 bacteria culture overnight (16 h) in LB medium using four 50 mL tubes for 5 min at 5000 rpm. Then discard the supernatant and wash the pellet twice with DPBS buffer pH 7.2 at 5000 rpm and finally resuspended it in lysis buffer (2%p/v SDS, 4% b-mercaptoethanol and tris-HCl pH 6.8 0.5 M) and incubate for 10 min at 100°C in a thermoblock. Then add 23 µL of DNAse buffer, 17 µL of DNAse I and incubate at 37°C for 30 min, followed by the addition of 20 µL of Proteinase K mixed by inversion, then incubate 4 h at 60°C incubation. In a gas extraction hood, add a 70°C preheated 90% phenol solution (90% phenol 0.1% b-mercaptoethanol and 0.2% 8-hydroxyquinoline) and incubate at 70°C for 15 min; it is worth noting that solution must be vortexed vigorously every 5 min during the incubation time. Then, incubate the solution at -20°C for 10 min, followed by centrifugation at 14000 rpm for 10 min. Next, the aqueous phase must be transferred into a clean tube, mixing vigorously with an ethyl ether saturated tris-EDTA solution. Then, centrifuge the samples for 1 min and discard the organic phase. A critical step required to eliminate contaminants and buffers is to perform extensive dialysis with 500–1000 Dalton membrane against ultrapure water at 25°C under stirring and periodical water exchange for at least 48 h until the residual phenol in the aqueous phase is completely removed. Finally, the samples can be concentrated by lyophilization or by centrifugation and vacuum for 2–3 h at 60°C, resuspend in 200 µL with D-PBS buffer and stored at -20°C. Alternatively, include an HPLC purification step using a C18 column at this time.

To quantify the LPS we recommend the 2-Keto-3-deoxyoctonate acid (Kdo) detection method using the Purpald reagent, followed by oxidation with sodium metaperiodate (NaIO4) [6]. This method quantifies the LPS content by comparison to a standard curve of commercially available LPS or Kdo. For this, prepare 5-fold serial dilutions (10^−3^ to 10^−4^) from previously extracted-LPS samples, and incubate with 50 µL NaIO_4_32 mM for 20 min to reduce the LPS Kdo groups. Next, add 50 µL of Purpald reagent 136 mM, and after 25 min incubation, add 50 µL NaIO_4_36 mM and incubate for another 20 min [6]. Then, the presence of aldehyde groups is recorded by the change in color of the Purpald reagent, measured at 550 nm in a microplate reader. The concentration determination is made using serial dilutions of Kdo. It is worth noting that concentration estimate depends on molar weight and the Kdo moieties of each LPS, from which the concentration of a reference compound is useful if Kdo in the LPS sample is not defined, i.e. the LPS from *E. coli* BW25113.

### Vesicle Preparation & characterization

The LPS-V can be made by the extrusion method as described elsewhere [7]. Briefly, quantified LPS's dissolved in 1 mL of D-PBS buffer pH 7.2 are heated above 60°C and extruded with a thermobarrel extruder (Avanti Polar) 10 times through two stacked 0.2 µm polycarbonate membranes (Whatman, Florham Park, NJ). Although the effects of storage – analyzed through DLS – appear to vary with each LPS-chemotype, the vesicles could be store for 12 h at 4°C, from which must be equilibrated at 25°C before analysis (Table ST2). LPS-V stability is 48 h maximum at 4°C. We recommend performing a size and polydispersity analysis (PDI) through Dynamic Light Scattering (DLS) [8]. Our protocol makes use of the Malvern Zetasizer Nano ZS equipment using D-PBS as a medium, using viscosity and refractive index of 0.8872 cP and 1330, respectively. It is worth noting that samples must be transferred to a polystyrene cuvette avoiding bubble formation samples and equilibrated at 25°C for 15 min before measuring. We recommend performing three measurements for each concentration of LPS-V, using 1 mL of the sample with 60 s of equilibration. It is important to note that different LPS-V concentrations should deliver a mean size lower than 0.2 µm and similar PDI to continue.

### LPS-V thermo-grams characterization by Differential Scanning Calorimetry (DSC)

A key component of this characterization protocol is the use of Differential Scanning calorimetry to check for sample reproducibility, which can be determined by examining the vesicles thermograms for anomalous peaks, transition temperature, or enthalpy changes. The protocol presented herein used the TA instruments NanoDSC calorimeter, which has a low volume cell, but the procedure is equivalent for other equipment such as the Malvern MicroCal calorimetry. Firstly, degas at least 2 mL of D-PBS solution for 15 min at 216 mmHg at 25°C. This solution will provide the baseline thermogram and buffer for sample preparation. The baseline is determined by injecting 700 ul of this solution into the sample and reference cells and carrying out at least two heating and cooling cycles, going 5 to 95°C and 95 to 5°C, respectively, at an increment rate of 1°C/min at a constant pressure of 3 atm. It is important to note that the scan rate could impact the DSC thermograms, for which we recommend exploring ranges of 0.5 to 2°C/min, accordingly to previous research (Table ST5). A better baseline might be obtained by multiple heating and cooling cycles, for which it is recommended to start this process the night before sample measurements. After signal equilibration, replace the buffer from the sample cell with the solution that contains the quantified LPS-V, which must be degassed under the same conditions used for the baseline solution. It is crucial to use the same buffer solution batch for the sample, baseline, and measurements. The sample thermogram can then be determined using two heating and cooling cycles, ranging from 5 to 90°C, taking care of possible crystallization events.

The thermograms obtained could be analyzed with the Nano-Analyze program from T.A Instruments or other data-fitting programs like Origin (Originlabs). It is worth noting that spurious signals can be decreased on the baseline determination process by spline-based smoothing, averaging or other methods [9,10].

**Fig. 2:**
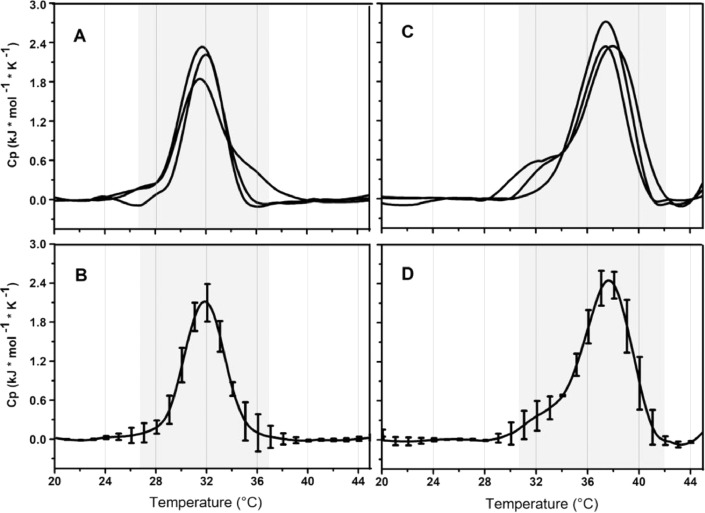
Thermo-grams of A) *E. coli* BW25113 *wild type* and C) Δ*waaP* mutant strain. B) and D) represent the mean and standard deviation respectively.

To describe each vesicle is useful to account for differences in heat capacities, melting temperature (Tm), width of the peak at half-height, transition ranges (Tr) and enthalpy of transition arising from variations of the LPS chemo-types used (Fig. 2). After baseline subtraction and peak limits recognition, fit a Gaussian function to calculate Tm's values, which corresponds to the temperature that correlates with the curve's peak, while Tr is the temperature range that covers the peak amplitude. Finally, perform a peak integration to calculate the calorimetric enthalpy (ΔH).

### Antimicrobial binding characterization by Isothermal titration calorimetry (ITC)

Micro-calorimetric measurements of heat of reaction are useful to characterize the antimicrobial binding to the LPS-V. One critical aspect of micro-calorimetry measurements is to avoid spurious effects arising from buffer mismatches, undesired protonation events, impurities, and sample heterogeneity. The latter can be avoided by prior DSC characterization selecting samples according to expected thermo-grams. To avoid buffer mismatch and impurities effects, we recommend exhaustively dialyzing the samples against the same buffer used for DSC calculations. Alternatively, use the same buffer of the DSC measurements to prepare the antimicrobial stock solution.

Herein we use a NanoITC isothermal titration calorimeter of TA instruments to characterize the LPS-V/Polymyxin B (PMB) binding isotherm at 25°C, but other antimicrobial solutions could be used. Firstly, degas 50 µL of 3 mg/mL [2.3 mM] PMB (antimicrobial solution) and 1.5 mL of D-PBS buffer for 15 min at 216 mmHg at 25°C. Use these solutions to determine the PMB heat of dilution on D-PBS by titrating 0.25 µL of the antimicrobial solution (titrant, syringe) on D-PBS (in the sample cell) every 5 min, under 200 rpm stirring at 25°C. The obtained thermogram serves as a baseline for further calculations. Then, we recommend washing twice the sample cell with buffer and degas the LPS-vesicle samples using the same parameters described above. The successful measurement of the heat of reaction by ITC depends on different factors, such as Kd between titrant and the macromolecule, reaction cell volume, injected volume, ΔH, and reaction stoichiometry (N). To obtain a more accurate estimate of Kd and ΔH, the ITC experiment must produce a thermogram with a sigmoid curve, for which the parameter C = [LPS] * Kd, must have a value between 10–100 [11]. As Kd between antimicrobials and LPS chemotypes is usually unknown, we recommend performing a macromolecular concentration gradient until an optimal titration isotherm is found to obtain the sigmoid curve. According to the sample cell volume of the TA Instrument Nano ITC, once the sample cell is clean, load 300 µL 0.125 mM, 0.25 mM, 0.5 mM and 1 mM LPS-V solutions and wait until the sample cell is equilibrated at 25°C. Then, titrate 0.25 µL of PMB [3 mg/mL] every 5 min with a stirring of 200 rpm. It is important to adjust the time between injections to complete the binding heat measurement. Between samples, wash the sample cell with 3 liters of ultrapure water followed by a final buffer-washing. The results analysis can be carried out through the NanoAnalyze software, free from T.A Instruments, or other software. For this, the LPS titration curve's baseline is subtracted, and the resulting thermogram is normalized and fitted into an independent site model.

## Method validation

It is well known that subtle LPS chemotype differences impact the LPS bilayer structure, stability, and bacterial sensibility to antimicrobials. For example, *E. coli wild-type* and Δ*waaP* isogenic mutant strains have LPS chemotypes that impact the OM's antimicrobial binding mechanism [12]. These chemotypes have the same hydrophobic acyl chains and different moieties at the inner core, thus represent a good model to analyze the ITC and DSC characterization sensibility.

We determined the thermotropic behavior of LPS-V and its binding isotherm to the antimicrobial Polymyxin B (PMB). Firstly, we extracted the LPS of *E. coli wild-type* and Δ*waaP* mutant strains, as their chemotype differences impact the LPS bilayer structure, stability and bacterial sensibility to PMB [13], [14]–15]. Accordingly, LPS extracted from three independent bacterial cultures were quantified by the Purpald method. As expected, each extraction procedure has a different LPS yield (Table ST1), hence the importance of quantifying and analyzing impurities and homogeneity in DSC. According to the methodology described above, after LPS quantification, vesicles were formed by extrusion and analyzed in DSC.

### LPS-V size and stability

Firstly, we extracted the LPS of *E. coli wild-type* and Δ*waaP* mutant strains from three independent bacterial cultures. As expected, each extraction procedure has a different LPS yield according (Table ST1), hence the importance of quantifying, and analyzing sample reproducibility later by DSC. According to the methodology described above, after LPS quantification, vesicles were formed by extrusion, and DLS was used to measure their size and polydispersity. Due to the LPS synthesis process, the vesicles obtained in this work are composed of more than one chemotype, however, the observed PDI values agree with those observed for vesicles obtained from commercial LPS of a single chemotype (Tables 1, ST5). As can be seen, the vesicle populations have comparable average diameter, below the 0.2 µm filter used. Interestingly, there is no explicit acceptable PDI value for LPS vesicles, however, most studies to date report values of 0.2 and 0.4, within the range described for lipid vesicle populations. [8,[16], [17], [18]–19]

### LPS-V thermo-gram analysis

Sample reproducibility can be observed through DSC. The normalized thermograms for each LPS containing vesicles are shown in Fig. 2. Although they are from different extractions, thermograms are consistent, with minor variations in their transition temperature (Tm). Furthermore, transition range (Tr) from gel to liquid crystalline, calculated as the width of the peak at half the height, agree with the one described for LPS-bilayer phases. According to their chemotypes differences, the average thermograms have Tm of 31.5°C and 37.7°C for *E. coli wild-type* and Δ*waaP* LPS-V respectively, indicating a greater restructuring of the LPS vesicle relative to the *wild-type* strain. It is important to observe that DSC thermograms are useful to characterize the effect of the subtle differences between samples of the Δ*waaP* LPS-V (Fig. 2C).

### Antimicrobial LPS binding characterization by ITC

**Fig. 3:**
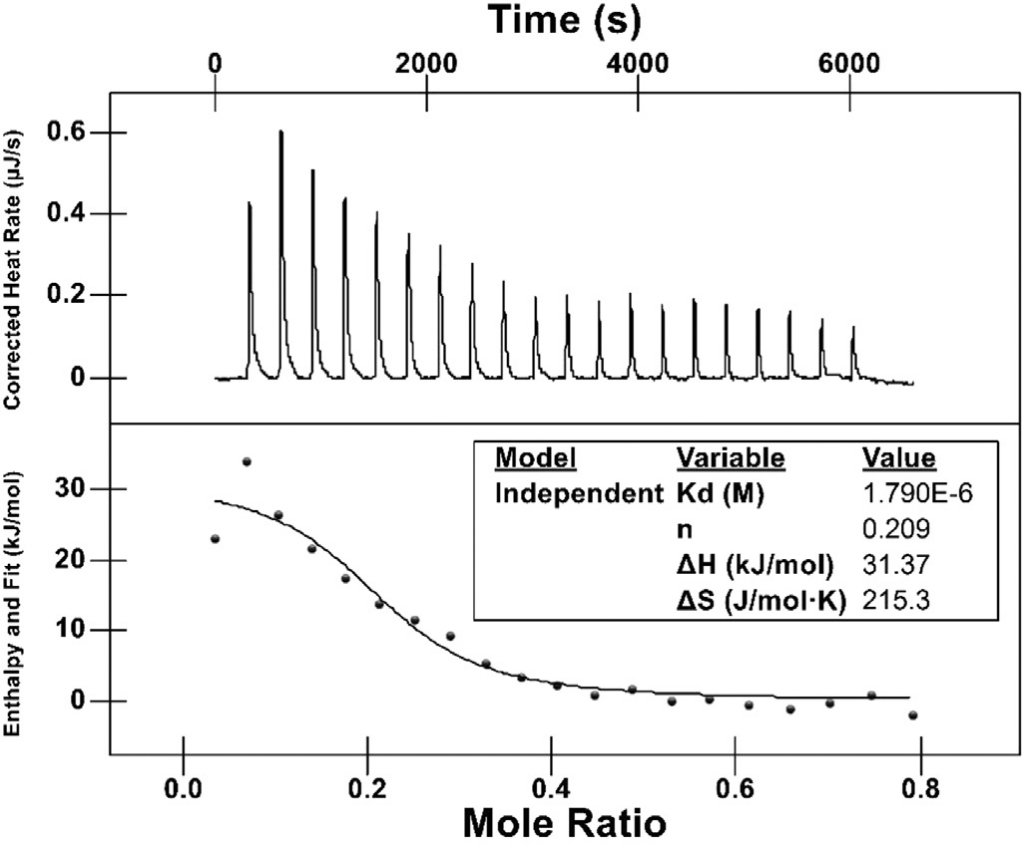
Heat profile and Isothermal calorimetric titration Analysis of LPS *wild-type* 1 mM from *E. coli* BW25113 with PMB 2.3 mM at 25°C.

**Fig. 4:**
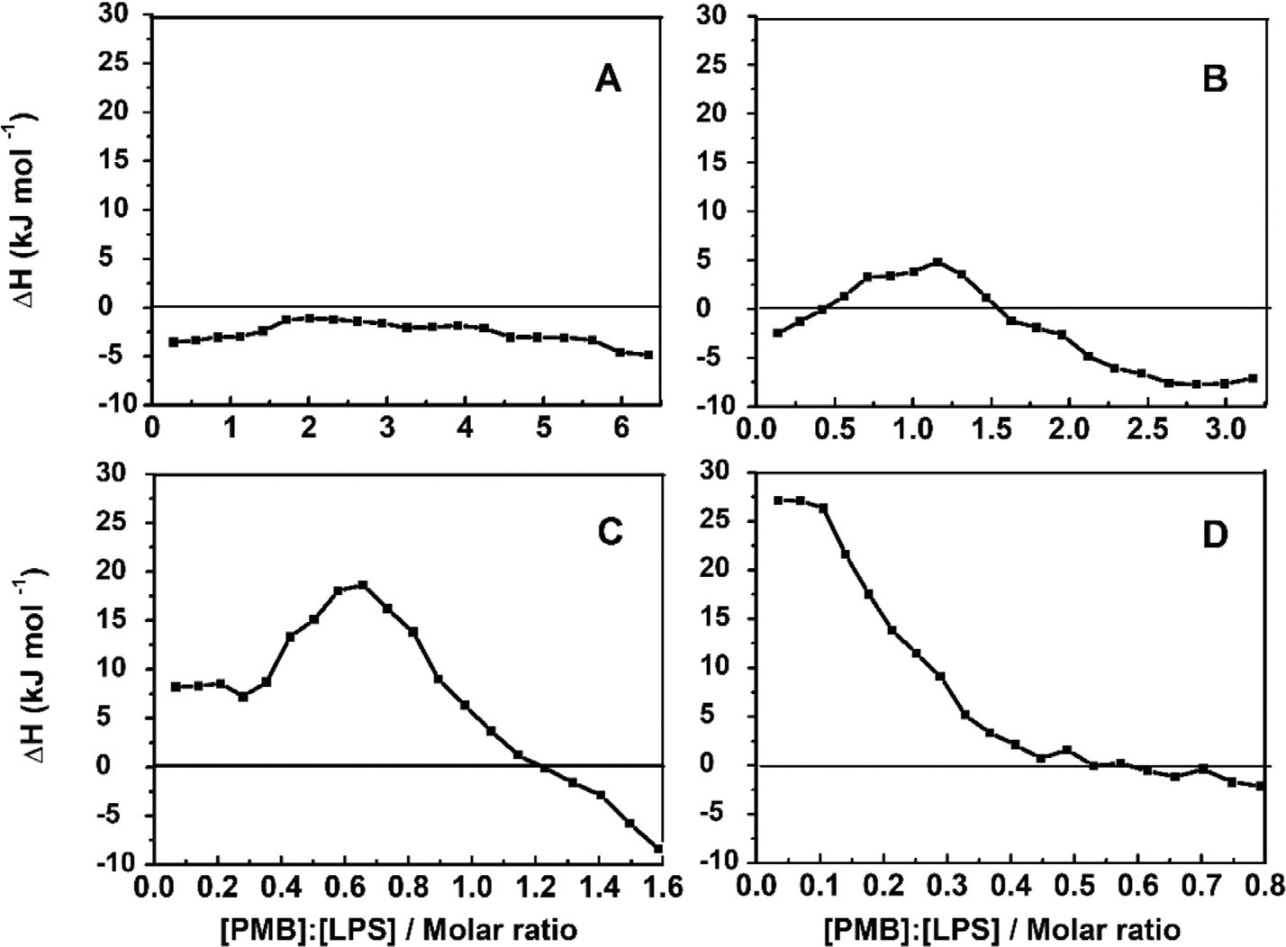
Binding isotherms of *E. coli wild-type* LPS-PMB (A) 0.125 mM, (B) 0.25 mM, (C) 0.5 mM and (D) 1 mM LPS-V concentrations at 25°C. For this, the LPS-V in the calorimetric cell was titrated every 5 min with 2.5 µL of PMB.

Reproducible DSC results of LPS-V samples allow characterizing the binding isotherm of different antimicrobials through ITC properly. Fig. 3 shows a representative heat profile (endothermic down) of PMB titration into the LPS-V containing solution. To obtain this profile, we followed the strategy of decreasing the reaction cell's LPS-V concentration to modulate the heat produced after each injection. Figs. 4 and S2 show the heat of reaction of a PMB 2.3mM solution titrated against LPS-V of *E. coli wild-type* and Δ*waaP* strains at 0.125, 0.25, 0.50, and 0.125 mM in the sample cell. At lower LPS-V concentration, the thermograms are flatter and not representative for the LPS-PMB binding (Fig. 3). Conversely, as the concentration of LPS-V increases, the thermograms adopt the expected sigmoid shape (Fig. 4D) Different thermogram profiles are displayed for each LPS chemotype assayed at the same concentration (Fig 4 and S2).  From this, it can be determined that PMB binds strongly to the Δ*waaP* LPS-V, in concordance with the *E. coli* strain sensitivity to this antimicrobial compared to the *wild-type* strain.

## Conclusion

The protocol presented herein complements DLS, DSC, and ITC methods to characterize how changes in the LPS chemotype of Gram-negative bacteria affect the supra-molecular behavior and antimicrobial binding mechanisms of LPS artificial vesicles. To isolate the LPS chemical moieties effects, the researchers use LPS extracted from different commercially available bacterial strains, which could produce artifacts due to LPS modifications arising from differential gene expression, thus a direct correlation within microbiological and biophysical assays is not possible. In contrast, we propose using LPS extracted from isogenic mutant strains to correlate the biophysical findings with microbiologic experiments made under the same growing conditions. It is worth noting that isogenic mutants can be obtained by today's routinary methods for different Gram-negative bacteria, allowing the expansion of the knowledge required to design novel antimicrobials. One key element of our protocol is DSC thermograms' use to corroborate the intra-sample reproducibility. This is reflected in Fig. 2 where each bacterial culture has almost the same Tm, indicating small internal changes within the transition such as counterion concentration differences, purification impurities, among others [20,21]. Despite subtle intra-sample variations are observed (Fig. 2C), its variability has a minor impact on the average thermogram's accuracy. Furthermore, in agreement with microbiological experiments that show a different hydrophobic core exposure between the *wild-type* and mutant strain, a major rearrangement of the LPS-vesicles is clearly visible on the thermograms. This protocol uses DSC as a tool to ensure sample reproducibility when the LPS is extracted from different bacterial cultures. This represents an advantage for assays that require higher concentrations or when LPS-chemotypes are not commercially available. Finally, this method is useful to find the LPS-concentration to perform thermodynamic analysis of antimicrobial binding to vesicles mimicking the bacterial outer membrane, as required to design novel antimicrobials Table 1.

**Table 1:** Size and PDI values of LPS-V. (n = 3)

Strain	|X| Size (d.nm)	|X| PDI value
*E. coli wild-type*	165 ± 24	0.202
*E. coli ΔwaaP*	105 ± 17	0.305

## Declaration of Competing Interest

The authors declare that they have no known competing financial interests or personal relationships that could have appeared to influence the work reported in this paper.

## Appendix Supplementary materials

Image, application 1
