# Supplementary material for: A method for characterizing the thermal stability and antimicrobial binding to Lipopolysaccharides of Gram-negative isogenic mutant strains
Source: MethodsX. 2021 Jul 30;8:101474. doi: 10.1016/j.mex.2021.101474 (PMC8374694; doi:10.1016/j.mex.2021.101474)
Supplement: Supplementary file 1 [file mmc1.docx]

**Supplementary material *and*  additional information:**

**Table ST1.** LPS concentrations, Tm, Size and PDI value obtained for each extraction and LPS-V.

| *Strain* | ***E. coli* *wild-type***  **(LPS molar weight 3813,7)** | | | ***E. coli* Δ*waaP***  **(LPS molar weight 3783,7)** | | |
| --- | --- | --- | --- | --- | --- | --- |
|  | ***Extraction 1*** | ***Extraction 2*** | ***Extraction 3*** | ***Extraction 1*** | ***Extraction 2*** | ***Extraction 3*** |
| LPS (mg/mL and mM) | 26.7 mg/mL  7.2 mM | 25.9 mg/mL  6.8 mM | 16.7 mg/mL  4.4 mM | 18.6 mg/mL  4.9 mM | 34.8 mg/mL  9.2 mM | 25.9 mg/mL  6.8 mM |
| Tm  (°C) | 30.9 | 31.6 | 31.9 | 38.1 | 37.9 | 36.9 |
| width half-heigth  (°C) | 2.3 | 3.1 | 2.4 | 4.2 | 3.7 | 4.2 |
| Size  (d.nm) | 164 | 190 | 142 | 91.3 | 91.3 | 122 |
| PDI | 0.198 | 0.242 | 0.167 | 0.314 | 0.280 | 0.322 |


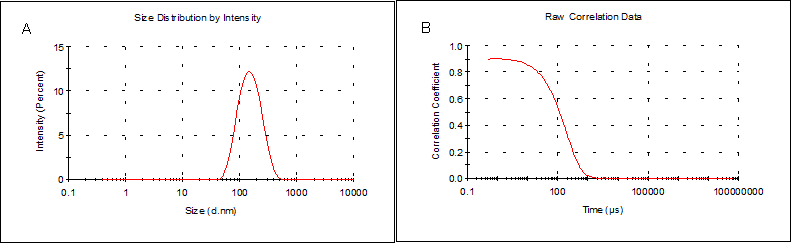
**Figure S1**. Size Distribution of the LPS vesicles. A) Size Distribution by Intensity, B) Raw correlation data. Graphics by dynamic light scattering (DLS).

**
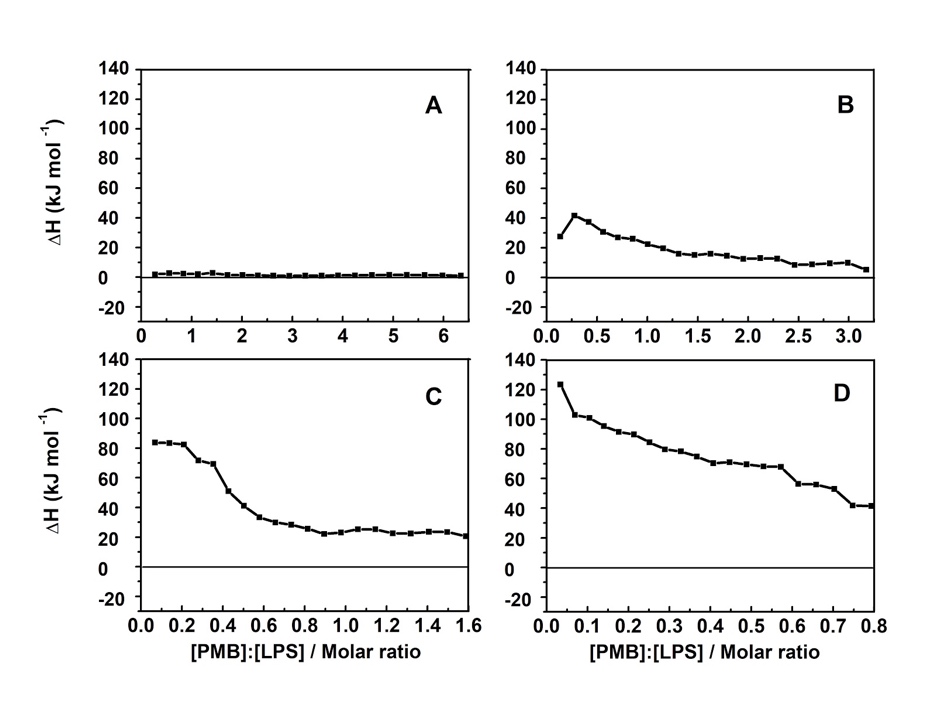
**

**Figure S2**. Binding isotherms *E. coli* Δ*waaP* LPS-peptide reaction for (A) 0.125mM, (B) 0.25mM, (C) 0.5mM and (D) 1mM LPS-V concentrations at 25 °C.

**Table ST2:** Storage Time at 4 °C effects on the PDI and Size values.

| Time Freeze (h) | 12 | 24 | 36 |
| --- | --- | --- | --- |
| PDI | 0.26 | 0.47 | 0.51 |
| Size (nm) | 186.3 | 202.7 | 271.2 |

**Table ST3:** Descriptions of strains used.

| **Strain** | **Name** | **Genotype** | **Source or reference** |
| --- | --- | --- | --- |
| BW25113 | *E. coli* | Δ(araD-araB)567, ΔlacZ4787(::rrnB-3), λ-, rph-1, Δ(rhaD-rhaB)568, hsdR514 | B.L. Wanner, CGSC cepa  N°7739, Keio collection |
| JW3605 | *E. coli* Δ*waaP* | Δ(araD-araB)567, ΔlacZ4787(::rrnB-3), λ-, ΔrfaP741::kan, rph-1, Δ(rhaDrhaB)568, hsdR514 | B.L. Wanner, strain CGSC  N°7739, Keio collection |

**Tables ST4**: Reagents and Equipment.

| **Assay** | **Reagents** | **Equipment** |
| --- | --- | --- |
| LPS extraction | DPBS(1X)(Sigma-Aldrich,D8537), SDS (Sigma-Aldrich, L3771). Phenol 96% (Winkler). Trizma-base (Sigma-Aldrich, T4661). EDTA (Sigma-Aldrich, EDS). 8-Hydroxyquinoline P.A. (Winkler). b-mercaptoethanol (Sigma-Aldrich, M3148)  Ultrapure water Type I, DNAse I 1 unit/μL (Sigma-Adrich, AMPD1), Proteinase K (Sigma-Aldrich, RPROTKSOL-RO).  Dialysis membrane 500-1000 Dalton(Applichem Darmstadt). |  |
| Purpald method | Sodium Metaperiodate (Sigma-Aldrich, S1878). Purpald (Sigma-Aldrich, 162892). 2-Keto-3-deoxyoctonate (Kdo) acid (Sigma-Aldrich, K2755). | Synergy H1 Hybrid Reader (Biotek Instruments, USA) |
| LPS Vesicles | Polycarbonate membrane with a pore diameter of 200 nm (Avanti Polar Inc). DPBS(1X)(Sigma-Aldrich, D8537). LPS samples (obtained in laboratory). | Mini-Extruder set (Avanti Polar Inc,USA) |
| Vesicles stability | LPS vesicles. Cuvette polystyrene.Ultrapure water Type I. | Malvern Zetasizer Nano ZS equipment (Malvern,UK) |
| Isothermal titration calorimetry (ITC) | LPS vesicles. Polymyxin B (Sigma-Aldrich, P4932). DPBS(1X)(Sigma-Aldrich, D8537), Ultrapure water Type I. | TA instruments NanoITC calorimeter. |
| Differential scanning calorimetry (DSC) | LPS vesicles. DPBS (1X)(Sigma-Adrich, P4932), Ultrapure water Type I. | TA instruments NanoDSC calorimeter. |

**Table ST5**: DSC parameters references

| **Paper DCS technique** | **Authors** | **DSC Scan rate**  °C/min | **PDI** | **Tr Range**  **°C** |
| --- | --- | --- | --- | --- |
| Aggregation Behavior of an Ultra-Pure Lipopolysaccharide that Stimulates TLR-4 Receptors. Biophys. J. 95, 986-993 (2008) | Sasaki H.  White S.H | 0.2 | 0.35 | 30-40 |
| Self-Organisation, Thermotropic and Lyotropic Properties of Glycolipids Related to their Biological Implications. Open Biochem J. 9, 49-72 (2015 | Garidel P. et al. | 1 | 0.40 | 30-40 |
| Effect of sodium deoxycholate sulfate on outer membrane permeability and neutralization of bacterial lipopolysaccharides by polymyxin B formulations. Chem. Phys. Lipids. 581, 119265 (2020 | Madhumanchi S. et al. | 1 | 0.2-0.5 | 20-35 |
| Biophysical characterization of the interaction of Limulus polyphemus endotoxin neutralizing protein with lipopolysaccharide. Eur. J. Biochem. 271, 2037-2046 (2004) | Andrä J. et al. | 1 | 0.5 | 25-40 |
| Structure of Supported Bilayers Composed of Lipopolysaccharides and Bacterial Phospholipids: Raft Formation and Implications for Bacterial Resistance. Biophys. J. 86, 3759-3771 (2004) | Tong J. et al. | 2 | - | 20-30 |
| Distortion of the lamellar arrangement of phospholipids by deep rough mutant lipopolysaccharide from Salmonella minnesota. J. Therm. Anal. Calorim. 82, 463-469 (2005) | Urbán E. et al. | 1 | - | 40 - 50  50 -64 |
| Investigation into the interaction of recombinant human serum albumin with Re-lipopolysaccharide and lipid A. J. Endotoxin. Res. 8, 115-126 (2002) | Jürgens G. et al. | 1 | - |  |
| Interaction of quorum signals with outer membrane lipids: insights into prokaryotic membrane vesicle formation. Mol. Biol. 69, 491-502 (2008) | Mashburn-Warren L. et al. | 1 | - | 20-40 |
| Low temperature thermal behaviour of lipopolysaccharides from Brucella and other Gram-negative bacteria. Thermochim. Acta. 215, 227-233 (1993) | Ramos M. et al. | 10 | - | - |
